# Maternal and Cord Blood Serum Metabolite Associations with Childhood Adiposity and Body Composition Outcomes

**DOI:** 10.3390/metabo13060749

**Published:** 2023-06-13

**Authors:** Monica E. Bianco, My H. Vu, James R. Bain, Michael J. Muehlbauer, Olga R. Ilkayeva, Denise M. Scholtens, Jami Josefson, William L. Lowe

**Affiliations:** 1 Ann & Robert H. Lurie Children’s Hospital of Chicago, Chicago, IL 60611, USA; mbianco@luriechildrens.org (M.E.B.); jjosefson@luriechildrens.org (J.J.); 2 Department of Pediatrics, Feinberg School of Medicine, Northwestern University, Chicago, IL 60611, USA; 3 Department of Preventive Medicine, Feinberg School of Medicine, Northwestern University, Chicago, IL 60611, USA; my_vu@alumni.brown.edu (M.H.V.); dscholtens@northwestern.edu (D.M.S.); 4 Duke Molecular Physiology Institute, Duke University School of Medicine, Durham, NC 27710, USA; james.bain@duke.edu (J.R.B.); michael.muehlbauer@duke.edu (M.J.M.); olga.ilkayeva@duke.edu (O.R.I.); 5 Department of Medicine, Division of Endocrinology, Metabolism and Nutrition, Duke University School of Medicine, Durham, NC 27710, USA; 6 Department of Medicine, Feinberg School of Medicine, Northwestern University, Chicago, IL 60611, USA

**Keywords:** maternal metabolites, childhood outcomes, offspring outcomes, cord blood metabolites, metabolomic, childhood adiposity

## Abstract

Maternal metabolites influence the size of newborns independently of maternal body mass index (BMI) and glycemia, highlighting the importance of maternal metabolism on offspring outcomes. This study examined associations of maternal metabolites during pregnancy with childhood adiposity, and cord blood metabolites with childhood adiposity using phenotype and metabolomic data from the Hyperglycemia and Adverse Pregnancy Outcome (HAPO) Study and the HAPO Follow-Up Study. The maternal metabolites analyses included 2324 mother–offspring pairs, while the cord blood metabolites analyses included 937 offspring. Multiple logistic and linear regression were used to examine associations between primary predictors, maternal or cord blood metabolites, and childhood adiposity outcomes. Multiple maternal fasting and 1 hr metabolites were significantly associated with childhood adiposity outcomes in Model 1 but were no longer significant after adjusting for maternal BMI and/or maternal glycemia. In the fully adjusted model, fasting lactose levels were negatively associated with child BMI z-scores and waist circumference, while fasting urea levels were positively associated with waist circumference. One-hour methionine was positively associated with fat-free mass. There were no significant associations between cord blood metabolites and childhood adiposity outcomes. Few metabolites were associated with childhood adiposity outcomes after adjusting for maternal BMI and glucose, suggesting that maternal BMI accounts for the association between maternal metabolites and childhood adiposity.

## 1. Introduction

Maternal obesity and gestational diabetes mellitus are associated with adverse pregnancy outcomes [1,2] and have an additive effect on birthweight (BW) and newborn adiposity [3,4,5]. The offspring of mothers with obesity and/or gestational diabetes have an increased risk of developing childhood obesity and/or impaired glucose tolerance [6,7,8,9]. It has been noted that maternal body mass index (BMI) and glycemia have both unique and shared metabolic signatures [10]. Maternal metabolites associated with maternal glycemia are enriched in gluconeogenic substrates, whereas maternal metabolites associated with maternal BMI are enriched in lipid-related metabolites [10]. Metabolic signatures associated with maternal BMI and glycemia contribute, in part, to the association of maternal BMI with newborn size at birth [11]. Recently, maternal fasting (triglycerides and several long-chain acylcarnitines) and 1 hr metabolites (branched-chain amino acids, proline, and alanine) were associated with newborn adiposity outcomes independently of maternal BMI and glucose [12]. While the mechanisms underlying the in utero risks for childhood obesity and dysglycemia are not well defined, it is hypothesized that fetal overnutrition in utero contributes to these risks [13,14].

Maternal amino acids, acylcarnitines, triglycerides, lipids, fatty acids, sugars, and their metabolites are associated with size at birth, newborn adiposity, and cord C-peptide independently of maternal BMI and glycemia [15,16]. This suggests a possibly important role in childhood outcomes. Our group previously examined associations between maternal and cord blood metabolites and newborn size at birth. Significant cord blood metabolite associations with birthweight include positive associations between cord blood branched-chain amino acids and medium-chain acylcarnitines and a negative association with 1,5-anhydroglucitol [17,18], with other groups reporting similar findings [19]. In addition to the cord blood associations, maternal alanine, a gluconeogenic precursor; nonesterified fatty acids; and triglycerides were associated with birthweight and/or the sum of skin folds (SSF) [16]. To date, no studies have examined the association of maternal and newborn metabolite levels with adiposity outcomes later in childhood. The objective of this study was to identify maternal and/or cord blood metabolites that are associated with childhood adiposity and body composition. Identifying a maternal and/or newborn metabolic signature associated with childhood obesity could allow for the identification of pregnant women and their neonatal offspring who would benefit most from targeted preventative interventions during pregnancy and/or early childhood.

## 2. Materials and Methods

### 2.1. Participants and Data and Sample Collection 

#### 2.1.1. Hyperglycemia and Adverse Pregnancy Outcome Study 

The HAPO Study was an international observational study conducted between 2000 and 2006 that recruited 25,505 participants [5]. Maternal blood samples were collected while the participants were fasting and after a 75 g glucose load during a 2 hr oral glucose tolerance test (OGTT) performed at 24–32 weeks of gestation, as previously described [5]. Maternal serum samples collected during the OGTT were processed and stored at −80 °C. Trained personnel measured standing height, weight, and blood pressure following standard procedures at time of the OGTT between 24–32 weeks [5,20,21]. Data on the participants’ age, gestational age at time of OGTT, family history of diabetes and hypertension, parity, and cigarette smoking and alcohol use during pregnancy were ascertained through a questionnaire. Cord blood was collected at delivery, and serum C-peptide was measured as previously described [22]. The HAPO protocol was approved by the Institutional Review Board of each field center, and written informed consent was given by each mother.

#### 2.1.2. Hyperglycemia and Adverse Pregnancy Outcome Follow-Up Study

Recruitment for the HAPO Follow-Up Study (FUS) occurred between 2013 and 2016 at 10 of 15 HAPO field centers worldwide [23]. Participants were recruited at 10–14 years of age using the following eligibility criteria: (a) caregivers and participants had to have been blind to HAPO OGTT results, (b) gestational age at delivery had to be 37 weeks or more, and (c) major neonatal malformations or fetal/neonatal death must not have occurred. At the HAPO FUS visit, the children’s demographic, anthropometric, and adiposity data were collected, as described previously [6,20,21,23,24]. For the measures of child anthropometrics, centralized training and maintenance of certification were conducted by the Clinical Coordinating Center [23].

Each child’s weight and height were measured twice to the nearest 0.1 kg and 0.5 cm, respectively, using calibrated scales. If results differed by >0.5 kg or >1.0 cm, a third measurement was obtained. Calibrated calipers (Harpenden, Baty International, West Sussex, UK) were used to measure skinfolds twice at three sites: triceps, subscapular, and suprailiac to the nearest 0.1 mm. If results differed by >1.0 mm, a third measurement was obtained [23]. Body composition was measured using air displacement plethysmography (Bod Pod, Cosmed, Albano Laziale, Italy), which provided data on fat mass and percentage of body fat. Each child’s age, first-degree family history of diabetes, and menstrual history (for girls) were collected from their mother via questionnaire.

### 2.2. Metabolomic Assays

Metabolomic assays were performed on maternal fasting and 1 hr sera samples from the HAPO OGTT. Maternal metabolomics data and childhood adiposity data were available for 2324 mother–offspring pairs from 10 field centers (Barbados, Belfast, Bellflower, Chicago, Cleveland, Hong Kong, Manchester, Toronto, Petah-Tiqva, and Bangkok). Exploratory targeted metabolomic assays were also performed on 937 newborn cord blood sera, only 461 of these cord blood samples had untargeted metabolomic data available.

#### 2.2.1. Conventional Metabolites and Targeted Metabolomic Assays

Sixty-two conventional clinical and targeted metabolites were quantitatively assayed, as previously described [15]. Briefly, levels of conventional clinical metabolites (lactate, triglycerides, 3-hydroxybutyrate, glycerol, and NEFA) were measured using a Unicel DxC 600 clinical analyzer (Beckman Coulter, Brea, CA, USA). Targeted metabolomic assays for acylcarnitines and amino acids were performed via tandem mass spectrometry (MS), for which known quantities of stable isotope-labelled internal standards were added using an Acquity TQD Triple Quadrupole system (Waters Corporation, Milford, MA, USA).

#### 2.2.2. Untargeted Assays

Untargeted gas chromatography (GC)–MS assays were performed to analyze a wide range of metabolites in serum, as described in [14]. Methanol, used as the extraction solvent, was spiked with a retention-time-locking (RTL) internal standard of perdeuterated myristic acid. Extracts were prepared for gas chromatography (GC)–MS using methoximation and trimethylsilylation [25,26]. Peaks were deconvoluted with AMDIS freeware [27] and parsed against the Fiehn RTL spectral library [26], with additions from our laboratory and elsewhere. Manual curation included selecting reliable peaks, which often represented isomers that we identified using common metabolite names [25]. Detected peak areas were log2-transformed for analysis. Fasting and 1 hr maternal serum sample pairs were batched for GC–MS, together with the offspring’s sera, when all three were analyzed. Quality control (QC) pools were constructed using equal volumes from all maternal samples and prepared for analysis as described above. QCs were injected as the first, middle, and last samples of each GC–MS batch. For cord blood samples, QC pools were constructed using small volumes from all cord blood samples, prepared as described above, and injected as the first, middle, and last samples of each daily GC/MS batch. To control technical variability attributable to batch and run order, GC–MS data were normalized using the QC data and our metabomxtr R package, version 1.16.0 [15,28].

### 2.3. Outcomes and Predictors

Childhood continuous outcomes included BMI, BMI z-score, fat mass, fat-free mass, percentage of body fat, waist circumference, and sum of three skinfolds (triceps, subscapular, and suprailiac crest). BMI z-scores were calculated using sex- and age-specific lambda–mu–sigma (LMS) curves [29]. SSF (mm) was calculated by summing the three skinfolds. Childhood dichotomous outcomes included obesity, SSF > 85th percentile, and body fat percent > 85th percentile. Maternal targeted and untargeted metabolites at fasting and 1 hr were treated as predictors of childhood adiposity outcomes. Cord blood metabolites were also analyzed as predictors of childhood adiposity outcomes.

### 2.4. Statistical Analysis

Continuous variables were summarized using means and SDs, and categorical variables were summarized using tables of frequencies and counts. Associations of metabolites (maternal fasting, 1 hr, and newborn cord blood) with continuous and categorical childhood anthropometric outcomes were examined using linear regression and logistic regression, respectively. Covariate adjustments were examined based on previous HAPO analyses and known potential confounders, as follows: model 1—field center (each with a high level of racial/ethnic homogeneity), child’s sex and age, gestational age at the time of OGTT, maternal age, mean arterial pressure, height, parity, smoking status, drinking status, and family history of diabetes at pregnancy during OGTT; model 2—model 1 + maternal BMI at pregnancy during OGTT; model 3—model 1 + maternal sum of glucose z-scores at pregnancy during OGTT; and model 4—model 1 + maternal BMI and sum of glucose z-scores at pregnancy during OGTT. *p*-values for all corresponding hypothesis tests were controlled with respect to false discovery rate (FDR) using Benjamini–Hochberg’s procedure [30]. FDR adjustment was performed for each outcome and each metabolomics sample type (maternal fasting, 1 hr and newborn cord blood). All analyses were conducted using R 4.1.2 software [31].

## 3. Results

### 3.1. Study Population

The characteristics of the mothers and their offspring included in the analyses examining the association between maternal metabolites and childhood outcomes are shown in Table 1. Table 2 displays these characteristics with regard to the analyses examining the association between cord blood metabolites and childhood outcomes. Mother–offspring pairs included in the analyses of maternal metabolites associated with childhood outcomes and those included in the analyses of cord blood metabolites were similar, except for small differences in race/ethnicity composition (notably, fewer mothers of White and Asian ancestry and more mothers of Mexican-American ancestry as a percentage of the cohort included in the analyses of cord blood metabolites) and in mean weight and percentage of male offspring at follow-up.

### 3.2. Maternal Metabolites

Initial analyses examined associations of maternal fasting and 1 hr metabolites with childhood adiposity outcomes (Table 3). Fasting lactose levels were negatively associated with child BMI z-score (β = −0.17, CI: −0.28–−0.06, *p* = 0.002) and waist circumference (iliac) (β = −1.85, CI: −2.94–−0.76, *p* = 0.001) in Models 1–4, while urea was positively associated with waist circumference (iliac) (β = 1.98, CI: 0.91–3.05, *p* < 0.001) in all four models. Fasting lactose levels were negatively associated with child fat mass and fat-free mass in Models 1 and 3, but the association was attenuated following adjustment for maternal BMI in Models 2 and 4. Other fasting metabolites, including maltose and 5-alpha-coprostanol, were also significantly associated with child fat-free mass and child fat mass in Models 1 and 3, but these associations were again attenuated after adjusting for maternal BMI. Of note, fasting urea levels were significantly associated with child BMI z-score, fat-free mass, and fat mass only after adjusting for maternal BMI in Models 2 and 4, while fasting aminomalonic acid levels were significantly associated with child BMI z-score in Models 2 and 4. Finally, fasting methionine was significantly associated with child fat mass in Models 1, 2, and 4.

In the analyses of maternal 1 hr metabolites (Table 4), methionine was positively associated with fat-free mass (kg) in Models 1–4 (β =0.47, CI: 0.22–0.71, *p* < 0.001, Model 4). A number of other 1 hr metabolites were significantly associated with child BMI z-score, fat-free mass, fat mass, and/or waist circumference in Models 1 and 3 but not after adjustment for maternal BMI in Models 2 and 4. These included leucine/isoleucine, tyrosine, glucose, lactose, glycerol, 3-hydroxybutyrate, and acetylcarnitine, as well as the low-abundance acylcarnitines, octenedioyl carnitine (AC C8:1 DC), dodecenoyl carnitine (AC C12:1), and docosanoyl carnitine (AC C22:0).

Due to the limited availability of targeted and untargeted metabolite data for cord blood samples, our analytical power with respect to identifying associations between cord blood metabolites and childhood outcomes was limited. Exploratory analyses were performed to examine these associations, and no significant associations between cord blood metabolites and childhood adiposity outcomes were found in any of the models (Supplemental Table S1).

## 4. Discussion

This study examined associations between maternal metabolites and childhood adiposity and body composition according to the HAPO cohort. Few strong associations were observed, with the notable exceptions being a negative association of maternal fasting lactose levels with offspring BMI z-score and waist circumference, a positive association of maternal fasting urea levels with waist circumference, and a positive association between methionine and child fat-free mass in the fully adjusted model. Interestingly, fasting aminomalonic acid and urea levels were significantly associated with child BMI Z-scores and fat mass, respectively, but only in Models 2 and 4, which included adjustments for maternal BMI. Among the analyzed maternal 1 hr metabolites, only methionine was positively associated with fat-free mass in the fully adjusted model. No significant associations between metabolites and other adiposity measures, including SSF, BMI, fat mass, and percentage of body fat, were found following adjustment for BMI. Although a number of fasting and 1 hr metabolites were significantly associated with child adiposity outcomes in the baseline model, most of these associations were attenuated after adjusting for maternal BMI.

Previous studies established associations of maternal and cord blood metabolites with newborn size and adiposity/composition, respectively [10,11,16,17,18,19], but the current study demonstrates that these associations did not persist through peripubertal childhood. The cord blood results are consistent with the results of a previous study that examined the association between cord blood kynurenine metabolites and birthweight. The study identified a positive association of kynurenine, quinolinic acid, and xanthurenic acid with birthweight; however, these associations did not persist to 4.5 years of age [32].

Levels of branched-chain amino acids (valine and leucine) have been shown to be significantly higher in obese compared to lean children [33,34]. A recent study also found that exposure to any fetal overnutrition was associated with changes in children’s metabolic profiles, including the sphingomyelin-mannose, skeletal muscle, and 3-carboxy-4-methyl-5-propyl-2-furanpropanoic acid profiles [35]. Our study demonstrated that the association between branched-chain amino acids and childhood obesity is not evident during pregnancy or at birth. Thus, this association develops at a later stage of development.

Our study is the first to examine associations of maternal and cord blood metabolites with childhood adiposity and body composition measurements obtained from children over a decade following their birth. Our finding that maternal lactose, urea, and methionine levels were associated with childhood body composition suggests that these metabolites may be reflective of the early-life origins of obesity. Adult studies have reported that high fasting methionine levels are associated with increased fat-free mass [36]. This is consistent with our finding that, in the fully adjusted model, maternal fasting and 1 hr methionine were associated with childhood fat-free mass. It is thought that methionine is a proteinogenic amino acid; however, the mechanism underlying its association with fat-free mass has not been elucidated. It was initially proposed that methionine stimulates the phosphorylation of the ribosomal protein S6 kinase(S6K1), leading to increased protein synthesis through the mTOR pathway [37,38]. However, multiple studies have shown that there is not a significant difference in the phosphorylation of S6K1/mTOR in methionine-restricted vs. methionine-supplemented animals. However, in birds, the supplementation of methionine resulted in increased breast muscle growth. Methionine supplementation may also decrease protein degradation through the inhibition of NF-κB, which is responsible for protein degradation [37].

Urea and lactose in maternal circulation have not been previously shown to be associated with adiposity or body composition in children or adults. The positive association between child waist circumference and maternal urea levels is consistent with a greater use of amino acids as metabolic fuels, complex changes in the hepatic or renal detoxification of waste nitrogen, or the slight impairment of the renal clearance of urea, suggesting a shift in fuel utilization [39]. Of note, in pregnant women on hemodialysis, lower blood urea nitrogen levels are associated with more favorable pregnancy outcomes, and higher maternal blood urea nitrogen levels could have a lasting negative impact on offspring adiposity [40]. In adults enrolled in the Framingham Heart Study, specific urea-cycle intermediates were associated with adiposity, ornithine was positively associated with BMI, and citrulline was negatively associated with BMI [41]. Lower circulating citrulline levels in nonpregnant obese humans have been described by other investigators but not consistently [39,42]. In this study, higher maternal levels of urea were associated with greater child waist circumference, but no significant maternal urea cycle intermediates were associated with child adiposity outcomes.

Lactogenesis occurs in the second half of a pregnancy and can be detected by an increase in plasma lactose concentrations [43]. However, the initiation of lactogenesis can occur at different times. Maternal metabolites were obtained at 28 weeks of gestation, and lower lactose levels at this time were associated with higher child BMI and waist circumference, suggesting that delayed or poor lactose production during pregnancy may be related to greater childhood adiposity.

### 4.1. Strength and Limitations

The longitudinal nature of the HAPO cohort is a strength of this study, while the multiethnic composition and geographic diversity of the cohort allowed for the identification of shared associations across ancestry groups and a decrease in the influence of environmentally related associations specific to different field centers. There are several limitations to this study. Information on maternal environment and lifestyle such as diet and physical activity were not available; these factors can potentially contribute to circulating metabolite levels, although, as noted, the participants in the HAPO cohort hailed from various environments worldwide. This study was a secondary analysis; thus, a power analysis was not performed a priori for these analyses. The small sample size for the cord blood analyses could have contributed to the null results due to insufficient power.

### 4.2. Clinical Significance

Together, these data suggest that while maternal BMI and glycemia during pregnancy are important contributors to the long-term risk of childhood obesity, maternal metabolites during pregnancy appear to have a minimal additive effect on childhood adiposity outcomes and largely reflect maternal obesity. Thus, it does not appear that characterizing the maternal metabolome will improve the early identification of newborns at risk for later obesity.

## 5. Conclusions

In conclusion, a limited number of maternal metabolites during pregnancy were associated with childhood adiposity/body composition outcomes independently of maternal glycemia and BMI, while cord blood metabolites were not associated with childhood adiposity outcomes. These findings suggest that while maternal metabolites during pregnancy are associated with newborn outcomes, these associations may not persist through childhood and thereby serve as possible biomarkers for the future risk of obesity in the offspring.

## Figures and Tables

**Table 1:** Maternal metabolite associations: characteristics of mothers during HAPO pregnancy OGTT and their children at follow-up.

Characteristics—Mothers (n = 2324)	Mean (SD)
Age during OGTT (yrs.)	29.6 (5.7)
Gestational Age at OGTT (wks.)	27.7 (1.7)
Height (cm)	161.4 (6.8)
Weight (kg)	72.2 (14.5)
Body Mass Index (BMI) (kg/m^2^)	27.7 (5.1)
Mean Arterial Pressure (mmHg)	80.5 (7.9)
Fasting Plasma Glucose (mg/dL)	81.2 (6.7)
1 hr Plasma Glucose (mg/dL)	133.9 (30.2)
2 hr Plasma Glucose (mg/dL)	112.0 (22.7)
Sum of glucose z-scores	0.1 (2.3)
	**N (%)**
Race/Ethnicity	
White, Non-Hispanic	629 (27.1)
Hispanic	359 (15.4)
Black, Non-Hispanic	629 (27.1)
Asian	699 (30.1)
Other	8 (0.3)
Any Prenatal Smoking	94 (4.0)
Any Prenatal Alcohol Use	157 (6.8)
Parity (any prior delivery > 20 weeks)	1208 (52.0)
**Characteristics—Children (n = 2324)**	
**At Follow-up**	**Mean (SD)**
Age (yrs.)	11.3 (1.1)
Height (cm)	148.5 (10.1)
Weight (kg)	43.7 (14.0)
BMI (kg/m^2^)	19.5 (4.6)
BMI z-score	0.5 (1.3)
	**N (%)**
Sex—Male	1158 (49.8)

**Table 2:** Cord blood metabolite associations: characteristics of mothers during HAPO pregnancy OGTT and their children at follow-up.

Characteristics—Mothers (n = 937)	Mean (SD)
Age during OGTT (yrs.)	28.9 (5.7)
Gestational Age during OGTT (wks.)	27.7 (1.9)
Height (cm)	160.3 (7.3)
Weight (kg)	72.4 (15.0)
Body Mass Index (BMI) (kg/m^2^)	28.2 (5.2)
Mean Arterial Pressure (mmHg)	81.3 (7.8)
Fasting Plasma Glucose (mg/dL)	82.1 (6.7)
1 hr Plasma Glucose (mg/dL)	136.0 (31.2)
2 hr Plasma Glucose (mg/dL)	112.8 (23.2)
Sum of glucose z-scores	0.3 (2.3)
	**N (%)**
Race/Ethnicity	
White, Non-Hispanic	239 (25.5)
Hispanic	239 (25.5)
Black, Non-Hispanic	247 (26.4)
Asian	212 (22.6)
Other	0 (0.0)
Any Prenatal Smoking	48 (5.1)
Any Prenatal Alcohol Use	69 (7.4)
Parity (any prior delivery > 20 weeks)	532 (56.8)
**Characteristics—Newborn (n = 937)**	**Mean (SD)**
Gestational age at birth (wk.)	39.8 (1.2)
Birth Weight (g)	3411.5 (478.5)
**Characteristics—Children (n = 937)**	
**At Follow-up**	**Mean (SD)**
Age (yrs.)	11.5 (1.0)
Height (cm)	149.3 (9.2)
Weight (kg)	45.7 (13.9)
BMI (kg/m^2^)	20.2 (4.8)
BMI z-score	0.7 (1.3)
	**N (%)**
Sex—Male	441 (47.1)

**Table 3:** Fasting maternal metabolite associations with childhood adiposity measures.

Metabolite (Class)	Model 1 Beta, (CI), *p*-Value	Model 2 (M BMI) Beta, (CI), *p*-Value	Model 3 (M Glucose) Beta, (CI), *p*-Value	Model 4 Beta, (CI), *p*-Value
Child BMI Z-Score				
Aminomalonic acid (AA)	0.16, (0.05–0.28), 0.005	0.19, (0.08–0.29), 0.001 *	0.16, (0.05–0.28), 0.005	0.19, (0.08–0.3), 0.001 *
Urea (AA)	0.17, (0.06–0.29), 0.003	0.19, (0.09–0.3), <0.001 *	0.17, (0.06–0.29), 0.003	0.19, (0.09–0.3), <0.001 *
Lactose (CHO)	−0.24, (−0.35–−0.13), 0 *	−0.17, (−0.28–−0.06), 0.002 *	−0.24, (−0.35–−0.13), <0.001 *	−0.17, (−0.28–−0.06), 0.002 *
Child Fat-Free Mass (kg)				
Urea (AA)	0.83, (0.26–1.39), 0.004	0.93, (0.41–1.46), 0.001 *	0.83, (0.26–1.39), 0.004	0.93, (0.41–1.46), 0.001 *
Lactose (CHO)	−1.05, (−1.61–−0.48), <0.001 *	−0.71, (−1.26–−0.17), 0.01	−1.06, (−1.62–−0.49), <0.001 *	−0.73, (−1.27–−0.19), 0.009
Maltose (CHO)	−0.85, (−1.39–−0.31), 0.002 *	−0.64, (−1.15–−0.13), 0.014	−0.85, (−1.39–−0.31), 0.002 *	−0.6, (−1.11–−0.08), 0.023
5-alpha-Coprostanol or similar oxysterol (Lipid)	−1.21, (−1.94–−0.48), 0.001 *	−0.47, (−1.18–0.25), 0.2	−1.26, (−2–−0.52), 0.001 *	−0.55, (−1.26–0.17), 0.134
Methionine (AA)	0.42, (0.17–0.67), 0.001 *	0.44, (0.2–0.68), <0.001 *	0.43, (0.18–0.68), 0.001	0.44, (0.2–0.68), <0.001 *
Child Fat Mass (kg)				
Urea (AA)	1.19, (0.43–1.95), 0.002	1.31, (0.58–2.04), <0.001 *	1.19, (0.43–1.95), 0.002	1.31, (0.58–2.04), <0.001 *
Lactose (CHO)	−1.56, (−2.31–−0.81), <0.001 *	−1.19, (−1.92–−0.45), 0.002	−1.55, (−2.3–−0.8), <0.001 *	−1.19, (−1.93–−0.45), 0.002
Child Waist Circumference (iliac (cm))				
Urea (AA)	1.79, (0.66–2.92), 0.002 *	1.98, (0.91–3.05), <0.001 *	1.8, (0.68–2.93), 0.002 *	1.98, (0.91–3.05), <0.001 *
Lactose (CHO)	−2.49, (−3.61–−1.37), <0.001 *	−1.85, (−2.94–−0.76), 0.001 *	−2.47, (−3.59–−1.34), <0.001 *	−1.85, (−2.94–−0.76), 0.001 *
Hypoxanthine (PUR/PYR)	1.84, (0.7–2.98), 0.002 *	1.47, (0.37–2.57), 0.009	1.82, (0.68–2.96), 0.002 *	1.47, (0.38–2.57), 0.009

* Denotes significant values. Abbreviations: CI = confidence interval; AA = amino acid; CHO = carbohydrate; PUR/PYR = purine/pyrimidine.

**Table 4:** 1 hr Maternal Metabolite Associations with Childhood Adiposity Measures.

Metabolite (Class)	Model 1 Beta, (CI), *p*-Value	Model 2 (M BMI) Beta, (CI), *p*-Value	Model 3 (M Glucose) Beta, (CI), *p*-Value	Model 4 Beta, (CI), *p*-Value
Child BMI z-score				
Leucine/Isoleucine (AA)	0.11, (0.06–0.17), <0.001 *	0.04, (−0.01–0.09), 0.085	0.1, (0.05–0.15), <0.001 *	0.04, (−0.01–0.09), 0.097
Glucose (CHO)	0.21, (0.1–0.33), <0.001 *	0.12, (0.01–0.23), 0.035	0.23, (0.11–0.35), <0.001 *	0.14, (0.03–0.26), 0.015
Child Fat-Free Mass (kg)				
Leucine/Isoleucine (AA)	0.5, (0.24–0.75), <0.001 *	0.21, (−0.04–0.46), 0.103	0.49, (0.23–0.75), <0.001 *	0.25, (0–0.5), 0.051
Methionine (AA)	0.59, (0.34–0.84), <0.001 *	0.46, (0.22–0.7), <0.001 *	0.59, (0.33–0.84), <0.001 *	0.47, (0.22–0.71), <0.001 *
Tyrosine (AA)	0.46, (0.2–0.71), <0.001 *	0.13, (−0.12–0.38), 0.296	0.45, (0.19–0.7), 0.001 *	0.15, (−0.1–0.39), 0.243
Lactose (CHO)	−1.05, (−1.63–−0.47), <0.001 *	−0.68, (−1.24–−0.12), 0.018	−1.07, (−1.65–−0.49), <0.001 *	−0.71, (−1.27–−0.15), 0.014
Child Fat Mass (kg)				
Glycerol (Lipid)	0.57, (0.25–0.89), 0.001 *	0.31, (0–0.62), 0.053	0.49, (0.16–0.81), 0.003 *	0.28, (−0.03–0.59), 0.08
AC C2	0.65, (0.32–0.98), <0.001 *	0.16, (−0.17–0.48), 0.339	0.53, (0.2–0.87), 0.002 *	0.12, (−0.21–0.44), 0.47
AC C12:1	0.65, (0.3–0.99), <0.001 *	0.18, (−0.15–0.52), 0.289	0.54, (0.19–0.89), 0.002 *	0.15, (−0.19–0.48), 0.392
AC C22	−0.52, (−0.86–−0.19), 0.002 *	−0.42, (−0.74–−0.1), 0.011	−0.52, (−0.85–−0.19), 0.002 *	−0.42, (−0.74–−0.1), 0.01
AC C8:1-DC	0.58, (0.25–0.92), 0.001 *	0.34, (0.01–0.66), 0.041	0.54, (0.21–0.87), 0.001 *	0.32, (0–0.64), 0.048
3-Hydroxybutyrate (Lipid)	0.74, (0.4–1.07), 0 *	0, (−0.33–0.34), 0.984	0.54, (0.19–0.89), 0.003 *	−0.09, (−0.44–0.26), 0.615
Child Waist Circumference (iliac (cm))				
Glucose (CHO)	1.97, (0.84–3.1), 0.001 *	1.11, (0–2.22), 0.05	1.99, (0.78–3.2), 0.001 *	1.24, (0.06–2.42), 0.04
Lactose (CHO)	−1.97, (−3.13–−0.81), 0.001 *	−1.23, (−2.36–−0.1), 0.033	−1.92, (−3.09–−0.76), 0.001 *	−1.23, (−2.36–−0.1), 0.033

* Denotes significant values. CI = confidence interval; AA = amino acid; AC = acylcarnitine; CHO = carbohydrate.

## Data Availability

The data presented in this study are available on request from the corresponding author. Metabolomic data and codes used for analyses will be made available by the authors on request. Data will soon be publicly available through Northwestern University’s DigitalHub.

## Supplementary Materials

The following supporting information can be downloaded at: https://www.mdpi.com/article/10.3390/metabo13060749/s1, Supplemental Table S1: Association of cord blood metabolites to child adiposity (using fully adjusted model).
