# Supplementary material for: Maternal and Cord Blood Serum Metabolite Associations with Childhood Adiposity and Body Composition Outcomes
Source: Metabolites. 2023 Jun 13;13(6):749. doi: 10.3390/metabo13060749 (PMC10302619; doi:10.3390/metabo13060749)
Supplement: Supplementary file 1 [file metabolites-13-00749-s001.zip › metabolites-2406224-supplementary.pdf]

Supplementary Materials:

Supplemental Table S1. Association of cord blood metabolites to child adiposity (using fully adjusted model)				
Cord Blood Metabolite	Child BMI-z score<br><br>Beta, (CI), p-value	Child Waist Circumference (iliac cm)<br>Beta, (CI), p-value	Child Fat Free mass (kg)<br>Beta, (CI), p-value	Child Fat Mass (kg)<br><br>Beta, (CI), p-value
Alanine	-0.04, (-0.12 - 0.04), 0.333	-0.48, (-1.25 - 0.3), 0.227	-0.34, (-0.72 - 0.04), 0.078	-0.12, (-0.64 - 0.4), 0.644
Arginine	-0.03, (-0.11 - 0.05), 0.516	-0.13, (-0.91 - 0.65), 0.744	-0.23, (-0.61 - 0.15), 0.227	-0.04, (-0.56 - 0.48), 0.877
Asparagine/Aspartic acid	-0.06, (-0.13 - 0.02), 0.151	-0.39, (-1.13 - 0.34), 0.296	-0.2, (-0.56 - 0.15), 0.265	-0.34, (-0.84 - 0.15), 0.173
Citrulline	-0.02, (-0.1 - 0.06), 0.58	0.01, (-0.75 - 0.77), 0.978	-0.11, (-0.48 - 0.26), 0.547	-0.05, (-0.56 - 0.46), 0.855
Glutamine/Glutamic acid	0, (-0.09 - 0.08), 0.981	0.02, (-0.81 - 0.86), 0.957	0.01, (-0.39 - 0.42), 0.953	0, (-0.56 - 0.55), 0.992
Glycine	0, (-0.08 - 0.08), 0.989	0.06, (-0.68 - 0.8), 0.874	-0.15, (-0.52 - 0.21), 0.404	0.14, (-0.36 - 0.64), 0.595
Glycerol	-0.02, (-0.1 - 0.05), 0.559	-0.23, (-0.99 - 0.53), 0.559	-0.18, (-0.55 - 0.19), 0.339	-0.27, (-0.78 - 0.24), 0.297
Histidine	0.05, (-0.03 - 0.13), 0.218	0.16, (-0.6 - 0.92), 0.681	-0.19, (-0.56 - 0.18), 0.308	0.18, (-0.33 - 0.69), 0.486
Lactate	-0.04, (-0.12 - 0.03), 0.258	-0.41, (-1.14 - 0.32), 0.271	-0.2, (-0.56 - 0.16), 0.277	-0.16, (-0.65 - 0.33), 0.517
Leucine/Isoleucine	0.01, (-0.07 - 0.09), 0.797	-0.16, (-0.94 - 0.61), 0.681	-0.13, (-0.51 - 0.25), 0.508	-0.04, (-0.56 - 0.48), 0.873
AC C2	0.01, (-0.07 - 0.1), 0.717	-0.15, (-0.94 - 0.64), 0.71	0.06, (-0.33 - 0.44), 0.765	-0.17, (-0.7 - 0.36), 0.535
AC C3	-0.04, (-0.12 - 0.04), 0.28	-0.5, (-1.26 - 0.26), 0.2	-0.21, (-0.58 - 0.16), 0.266	-0.44, (-0.96 - 0.07), 0.089
AC C4/Ci4	-0.07, (-0.15 - 0.01), 0.106	-0.6, (-1.37 - 0.18), 0.134	-0.29, (-0.67 - 0.09), 0.136	-0.43, (-0.95 - 0.09), 0.108
AC C5:1	0, (-0.07 - 0.08), 0.912	-0.08, (-0.81 - 0.66), 0.839	0.04, (-0.32 - 0.4), 0.838	0.02, (-0.47 - 0.52), 0.922
AC C5	-0.02, (-0.09 - 0.06), 0.702	-0.36, (-1.12 - 0.4), 0.355	-0.13, (-0.5 - 0.24), 0.502	-0.32, (-0.83 - 0.19), 0.224
AC C4-OH	0.01, (-0.07 - 0.09), 0.853	0.12, (-0.67 - 0.91), 0.77	-0.03, (-0.41 - 0.36), 0.883	0.13, (-0.4 - 0.66), 0.623
AC C6	-0.08, (-0.27 - 0.11), 0.422	-0.93, (-2.66 - 0.8), 0.295	-0.58, (-1.45 - 0.28), 0.188	-0.46, (-1.61 - 0.69), 0.431
AC C5-OH/C3-DC	-0.11, (-0.24 - 0.02), 0.099	-1.09, (-2.31 - 0.12), 0.079	-0.56, (-1.13 - 0.02), 0.058	-0.63, (-1.46 - 0.21), 0.145
AC C4-DC/Ci4-DC	-0.01, (-0.1 - 0.09), 0.915	-0.15, (-1.09 - 0.78), 0.746	-0.1, (-0.54 - 0.34), 0.654	0.06, (-0.57 - 0.69), 0.853
AC C8:1	0.04, (-0.04 - 0.12), 0.362	0.24, (-0.58 - 1.06), 0.57	0.21, (-0.19 - 0.61), 0.301	0.24, (-0.31 - 0.79), 0.399
AC C8	-0.01, (-0.08 - 0.07), 0.878	-0.02, (-0.78 - 0.74), 0.958	-0.15, (-0.52 - 0.22), 0.44	0.17, (-0.33 - 0.68), 0.503
AC C5-DC	-0.01, (-0.09 - 0.07), 0.868	-0.12, (-0.91 - 0.66), 0.758	-0.11, (-0.49 - 0.27), 0.574	-0.11, (-0.64 - 0.42), 0.692
AC C6-DC/C8-OH	0, (-0.08 - 0.07), 0.907	-0.05, (-0.79 - 0.7), 0.906	0.19, (-0.18 - 0.55), 0.312	-0.03, (-0.53 - 0.47), 0.913

AC C10:3	0.02, (-0.06 - 0.1), 0.611	0.11, (-0.7 - 0.91), 0.792	-0.03, (-0.42 - 0.37), 0.897	0.17, (-0.37 - 0.71), 0.54
AC C10:2	0.01, (-0.07 - 0.09), 0.809	-0.02, (-0.79 - 0.75), 0.954	0.06, (-0.31 - 0.44), 0.747	0.08, (-0.43 - 0.6), 0.751
AC C10:1	0.01, (-0.07 - 0.09), 0.805	0.01, (-0.77 - 0.79), 0.982	-0.15, (-0.53 - 0.23), 0.436	0.06, (-0.46 - 0.59), 0.813
AC C10	-0.06, (-0.22 - 0.09), 0.437	-0.43, (-1.81 - 0.95), 0.541	0, (-0.67 - 0.67), 0.995	-0.21, (-1.13 - 0.71), 0.655
AC C10-OH/C8-DC	-0.01, (-0.09 - 0.07), 0.82	0, (-0.78 - 0.79), 0.99	0.18, (-0.2 - 0.56), 0.358	-0.04, (-0.57 - 0.49), 0.884
AC C12:1	0.01, (-0.07 - 0.09), 0.884	0.03, (-0.76 - 0.82), 0.933	-0.08, (-0.46 - 0.31), 0.7	0.34, (-0.19 - 0.87), 0.211
AC C12	-0.06, (-0.14 - 0.02), 0.124	-0.58, (-1.35 - 0.2), 0.146	-0.23, (-0.61 - 0.15), 0.232	-0.17, (-0.69 - 0.36), 0.537
AC C12-OH/C10-DC	0.02, (-0.06 - 0.09), 0.686	0.43, (-0.33 - 1.2), 0.269	0.32, (-0.05 - 0.7), 0.09	0.43, (-0.09 - 0.94), 0.104
AC C14:2	-0.03, (-0.11 - 0.05), 0.446	-0.35, (-1.11 - 0.41), 0.37	-0.27, (-0.65 - 0.1), 0.149	0.06, (-0.45 - 0.57), 0.817
AC C14:1	-0.01, (-0.09 - 0.07), 0.889	-0.13, (-0.9 - 0.65), 0.744	-0.11, (-0.49 - 0.27), 0.571	0.17, (-0.36 - 0.69), 0.532
AC C14	0.02, (-0.06 - 0.09), 0.701	0, (-0.77 - 0.76), 0.99	-0.04, (-0.41 - 0.34), 0.847	0.24, (-0.28 - 0.76), 0.366
AC C14:1-OH	0.07, (-0.02 - 0.16), 0.132	0.54, (-0.31 - 1.39), 0.212	0.37, (-0.04 - 0.78), 0.074	0.49, (-0.07 - 1.06), 0.086
AC C14-OH/C12-DC	0.03, (-0.06 - 0.12), 0.531	0.15, (-0.71 - 1), 0.738	0.21, (-0.19 - 0.62), 0.298	0.22, (-0.34 - 0.78), 0.445
AC C16	0.01, (-0.07 - 0.09), 0.719	-0.02, (-0.8 - 0.76), 0.956	-0.15, (-0.53 - 0.23), 0.444	0.25, (-0.28 - 0.77), 0.352
AC C16-OH/C14-DC	0.01, (-0.07 - 0.09), 0.869	0.08, (-0.69 - 0.84), 0.845	0.07, (-0.31 - 0.44), 0.724	0.23, (-0.28 - 0.74), 0.381
AC C18:2	0.03, (-0.05 - 0.11), 0.487	0.05, (-0.72 - 0.82), 0.894	-0.22, (-0.6 - 0.15), 0.245	0.21, (-0.3 - 0.73), 0.418
AC C18:1	-0.01, (-0.09 - 0.07), 0.773	-0.12, (-0.91 - 0.66), 0.759	-0.11, (-0.49 - 0.28), 0.584	0.16, (-0.37 - 0.68), 0.558
AC C18	0.09, (0 - 0.17), 0.038	0.79, (0 - 1.57), 0.05	0.22, (-0.16 - 0.61), 0.252	0.63, (0.1 - 1.15), 0.02
AC C18:1-OH/C16:1-DC	-0.02, (-0.1 - 0.07), 0.687	-0.11, (-0.91 - 0.68), 0.781	-0.09, (-0.47 - 0.29), 0.649	0.05, (-0.48 - 0.58), 0.86
AC C18-OH/C16-DC	0.02, (-0.06 - 0.1), 0.675	0.2, (-0.58 - 0.98), 0.615	0.08, (-0.3 - 0.46), 0.681	0.27, (-0.26 - 0.79), 0.319
AC C20	-0.02, (-0.1 - 0.07), 0.701	-0.13, (-0.92 - 0.66), 0.748	0.06, (-0.32 - 0.45), 0.744	-0.21, (-0.74 - 0.31), 0.425
AC C18:1-DC	0.01, (-0.07 - 0.09), 0.867	0.23, (-0.54 - 0.99), 0.561	-0.08, (-0.45 - 0.3), 0.687	0.34, (-0.18 - 0.85), 0.202
AC C20-OH/C18-DC	0.06, (-0.02 - 0.14), 0.134	0.7, (-0.05 - 1.46), 0.068	0.4, (0.04 - 0.77), 0.031	0.52, (0.01 - 1.02), 0.046
AC C22	0.02, (-0.06 - 0.11), 0.565	0.16, (-0.65 - 0.96), 0.7	-0.19, (-0.6 - 0.21), 0.354	0.13, (-0.4 - 0.67), 0.624
AC C8:1-OH/C6:1-DC	-0.03, (-0.1 - 0.05), 0.528	-0.16, (-0.94 - 0.61), 0.68	-0.09, (-0.47 - 0.29), 0.632	-0.16, (-0.68 - 0.36), 0.558
AC C7-DC	0.03, (-0.18 - 0.25), 0.751	0.73, (-1.19 - 2.65), 0.456	0.32, (-0.63 - 1.26), 0.512	0.4, (-0.85 - 1.66), 0.53
AC C8:1-DC	-0.02, (-0.1 - 0.07), 0.711	-0.01, (-0.8 - 0.78), 0.977	-0.08, (-0.46 - 0.31), 0.702	0.02, (-0.51 - 0.56), 0.936
AC C16:2	-0.04, (-0.15 - 0.07), 0.509	-0.08, (-1.11 - 0.94), 0.872	0.04, (-0.48 - 0.55), 0.889	0.12, (-0.55 - 0.79), 0.726

AC C16:1	-0.04, (-0.11 - 0.04), 0.38	-0.33, (-1.1 - 0.43), 0.394	-0.28, (-0.65 - 0.1), 0.146	0.13, (-0.38 - 0.64), 0.619
AC C16:1-OH/C14:1-DC	-0.01, (-0.09 - 0.06), 0.738	-0.07, (-0.83 - 0.69), 0.859	-0.17, (-0.54 - 0.2), 0.373	0.18, (-0.33 - 0.7), 0.481
AC C18:2-OH	0.05, (-0.04 - 0.15), 0.282	0.75, (-0.17 - 1.66), 0.11	0.13, (-0.32 - 0.57), 0.57	0.51, (-0.09 - 1.11), 0.098
AC C20:4	-0.02, (-0.1 - 0.06), 0.646	-0.18, (-0.98 - 0.62), 0.658	0, (-0.4 - 0.4), 0.996	-0.15, (-0.69 - 0.39), 0.593
3-Hydroxybutyrate	0.02, (-0.06 - 0.1), 0.666	0.38, (-0.4 - 1.16), 0.338	0.19, (-0.19 - 0.57), 0.325	0.26, (-0.26 - 0.78), 0.322
Methionine	-0.06, (-0.13 - 0.02), 0.15	-0.58, (-1.32 - 0.17), 0.131	-0.37, (-0.74 - -0.01), 0.045	-0.19, (-0.69 - 0.32), 0.469
NEFA	-0.01, (-0.08 - 0.07), 0.875	0.12, (-0.63 - 0.88), 0.749	-0.07, (-0.43 - 0.3), 0.721	0.29, (-0.21 - 0.8), 0.259
Ornithine	-0.07, (-0.15 - 0.01), 0.077	-0.61, (-1.39 - 0.17), 0.124	-0.46, (-0.84 - -0.08), 0.018	-0.31, (-0.83 - 0.22), 0.251
Phenylalanine	-0.06, (-0.14 - 0.02), 0.113	-0.51, (-1.29 - 0.26), 0.194	-0.41, (-0.79 - -0.04), 0.032	-0.18, (-0.7 - 0.34), 0.502
Proline	-0.05, (-0.12 - 0.03), 0.213	-0.58, (-1.32 - 0.17), 0.128	-0.52, (-0.88 - -0.16), 0.005	-0.08, (-0.58 - 0.42), 0.756
Serine	0.03, (-0.05 - 0.11), 0.486	0.08, (-0.69 - 0.84), 0.839	-0.15, (-0.52 - 0.23), 0.444	0.22, (-0.29 - 0.73), 0.403
Triglycerides	0, (-0.08 - 0.08), 0.927	-0.07, (-0.84 - 0.7), 0.859	-0.04, (-0.41 - 0.34), 0.843	0.03, (-0.48 - 0.55), 0.894
Tyrosine	-0.08, (-0.16 - 0), 0.058	-0.73, (-1.51 - 0.04), 0.063	-0.44, (-0.82 - -0.06), 0.022	-0.21, (-0.73 - 0.31), 0.429
Valine	0, (-0.08 - 0.08), 0.952	-0.18, (-0.95 - 0.6), 0.656	-0.08, (-0.46 - 0.3), 0.669	-0.09, (-0.61 - 0.43), 0.742
